# Troponin Aptamer on an Atomically Flat Au Nanoplate Platform for Detection of Cardiac Troponin I

**DOI:** 10.3390/nano10071402

**Published:** 2020-07-18

**Authors:** Hyoban Lee, Hyungjun Youn, Ahreum Hwang, Hyunsoo Lee, Jeong Young Park, Weon Kim, Youngdong Yoo, Changill Ban, Taejoon Kang, Bongsoo Kim

**Affiliations:** 1 Department of Chemistry, KAIST, Daejeon 34141, Korea; stban1829@gmail.com (H.L.); ahreumh@kaist.ac.kr (A.H.); hsoolee@kaist.ac.kr (H.L.); jeongypark@kaist.ac.kr (J.Y.P.); 2 Department of Chemistry, POSTECH, Pohang 37673, Korea; yhj1005@postech.ac.kr; 3 Bionanotechnology Research Center, KRIBB, Daejeon 34141, Korea; 4 Center for Nanomaterials and Chemical Reactions, IBS, Daejeon 34141, Korea; 5 Division of Cardiology, Department of Internal Medicine, Kyung Hee University Hospital, Kyung Hee University, Seoul 02447, Korea; mylovekw@hanmail.net; 6 Department of Chemistry, Ajou University, Suwon 16499, Korea; yyoo@ajou.ac.kr

**Keywords:** cTnI, aptamer, nanoplate, surface-enhanced Raman scattering

## Abstract

Well-ordered bioreceptors on atomically flat Au surfaces can be a high-performance biosensor. Cardiac troponin I proteins (cTnIs) have been regarded as a specific biomarker for acute myocardial infarction (AMI). Here, we report the accurate detection of cTnIs using an aptamer-immobilized Au nanoplate platform. The single-crystalline and atomically flat Au nanoplate was characterized by atomic force microscopy. For the precise detection of cTnI, we immobilized an aptamer that can strongly bind to cTnI onto an atomically flat Au nanoplate. Using the aptamer-immobilized Au nanoplate, cTnIs were successfully detected at a concentration of 100 aM (2.4 fg/mL) in buffer solution. Furthermore, cTnIs in serum could be identified at a concentration of 100 fM (2.4 pg/mL). The total assay time was ~7 h. Importantly, the aptamer-immobilized Au nanoplate enabled us to diagnose AMI patients accurately, suggesting the potential of the present method in the diagnosis of AMI.

## 1. Introduction

Acute myocardial infarction (AMI) is a common cause of death globally [1]. According to the Centers for Disease Control and Prevention (CDC), someone in the United States has a heart attack every 40 s [2]. Cardiac troponin I proteins (cTnIs) have been regarded as specific biomarkers for AMI because cTnIs are released only when myocardial tissues are damaged [1,3]. Therefore, diverse methods have been developed for the sensitive and specific sensing of cTnIs [4,5,6,7,8,9,10,11,12,13,14,15,16]. Typically, cTnIs have been identified by radioimmunoassay and enzyme-linked immunosorbent assay (ELSIA) [6,7]. However, these methods often suffer from limitations such as low sensitivity, poor stability, and the high cost of antibodies. Although several approaches have been recently developed for AMI biomarkers [8,9,10,11,12,13,14,15,16], there is still a need to improve sensing performance for detecting very low levels of cTnIs.

Atomically flat surfaced nanomaterials have been interfaced with various kinds of systems, improving nano-devices remarkably [17,18,19,20,21,22,23]. For the development of high-performance biosensors, it is important to prepare bioactive surfaces that can provide the maximized functionality of bioreceptors [23]. Well-ordered bioreceptors on atomically flat surfaces can exhibit improved affinity and specificity toward target species, thus they can be important building blocks in the realization of high-performance biological sensors [17,18,19,20,21,22]. To obtain an extremely sensitive and selective cTnI sensor, we combined an advanced bioreceptor of cTnI with an atomically flat Au (111) surface of a nanoplate. Previously, our group reported that the single-crystalline Au nanoplates have ultraflat and ultraclean surfaces [17] and thus biomolecules could be uniformly immobilized on the atomically flat Au nanoplates [17,18,19]. Additionally, aptamers, oligonucleic acids with high affinity and specificity toward target molecules, have been considered as outstanding bioreceptors because they have the beneficial properties of rapid production, high stability, and easy functionalization [4,5,24]. We also discovered a novel aptamer that can bind to cTnI 77 times more strongly than a commercial antibody through the systematic evolution of ligands by the exponential enrichment (SELEX) method [4,5]. Taken together, it is hypothesized that the combination of atomically flat Au nanoplates and a cTnI aptamer with high affinity can improve the sensing performance of cTnI.

Herein, we demonstrate that the optimal immobilization of an aptamer onto the atomically flat Au nanoplates can be a high-performance biosensor for cTnI. The atomic force microscopy (AFM) results show that the aptamer is well immobilized on the Au nanoplate and also provides evidence that the cTnIs can be captured on the aptamer-immobilized Au nanoplate. In addition, aptamer-functionalized Au nanoparticles (NPs) are employed as reporters for surface-enhanced Raman scattering (SERS)-based cTnI detection. Only in the presence of cTnIs, the Au NPs are assembled onto the Au nanoplate and an NPs-on-nanoplate structure is formed. Scanning electron microscopy (SEM) images clearly show the cTnI-detected NPs-on-nanoplate architectures. Moreover, we quantitatively detected cTnIs by measuring SERS signals from the NPs-on-nanoplate structures. As a result, cTnIs in buffer solution are detectable at a low concentration of 100 aM (2.4 fg/mL), and cTnIs in human serum can be identified at a low concentration of 100 fM (2.4 pg/mL). Most importantly, the well-ordered aptamer on the Au nanoplates enabled us to diagnose nine AMI patients accurately. Based on these results, we insist that well-immobilized bioreceptors on Au nanoplates will be useful for the diagnosis of several diseases.

## 2. Materials and Methods

### 2.1. Synthesis of Au Nanoplates

Atomically flat Au nanoplates were synthesized by using a horizontal hot-wall furnace system with a quartz tube. The alumina boat containing an Au slug (Sigma-Aldrich, St. Louis, MO, USA) was placed at the heating zone and the sapphire substrates were placed in the low-temperature zone. After the flowing of Ar gas for 30 min, the system was heated to 1100 °C and reacted for 60 min. The pressure of the system was kept as 10 Torr with an Ar gas flow rate of 100 sccm. After the reaction, atomically flat Au nanoplates could be obtained on the sapphire substrates.

### 2.2. Aptamer Sequences

Table 1 shows the aptamer sequences used in this experiment (COSMO Genetech, Seoul, Korea). The probe aptamer has a thiol group at the 3′-terminus. The reporter aptamer has a Raman dye (Cy5) at the 5′-terminus and a thiol group at the 3′-terminus, respectively. The SELEX method for discovering troponin aptamers was initiated with a single-stranded DNA (ssDNA) library consisting of random sequences of 40 nucleotides. The troponin aptamers were selected by His-tag capture magnetic beads. The binding percent of ssDNAs increased up to 63% in the 11th round but did not increase thereafter. The selected ssDNAs were, therefore, eluted after the 11th round and amplified by polymerase chain reaction.

### 2.3. Aptamer Immobilization on Au Nanoplates

As-synthesized Au nanoplates were transferred onto a Si wafer and incubated in a 1 µM probe aptamer solution (1 mL; 0.1 M phosphate-buffered saline (PBS), 10 mM NaCl, 5 mM KCl, 1 mM MgCl2, pH 7.4) at room temperature for 12 h. Next, the aptamer-immobilized nanoplates were rinsed with SELEX buffer (300 mM NaCl, 50 mM KCl, 10 mM MgCl_2_, 50 mM Tris-HCl, pH 8.3) and distilled deionized water.

### 2.4. Detection of cTnIs Using Aptamer-Immobilized Au Nanoplates

The cTnIs were added to a hybridization buffer solution (300 mM NaCl, 20 mM Tris-HCl, 0.5 mM beta-mercaptoethanol, 20% (*w/v*) glycerol, 0.05% Tween 20, pH 8.0) or a hybridization buffer solution with 20% human serum (Sigma-Aldrich). Au NPs (~10 nm) were purchased from Sigma-Aldrich and used after centrifugation (10,000× *g*, 30 min). Au NPs were dispersed in a final 500 µL of 1 µM reporter aptamer solution (0.05 M PBS, 5 mM NaCl, 2.5 mM KCl, 0.5 mM MgCl_2_, pH 7.4) at room temperature for 3 h. To remove excess reporter aptamers, the Au NP solution was centrifuged (10,000× *g*, 15 min), the supernatant was removed, and resuspended in PBS (500 µL). For the detection of cTnIs, the prepared cTnI solution (1 mL) was added to the aptamer-immobilized Au nanoplates at 35 °C. After 6 h, the Au nanoplates were washed with the hybridization buffer and distilled deionized water. Next, the cTnI-captured Au nanoplates were incubated in the Au NP reporter solution at 35 °C for 30 min. Finally, the cTnI-detected Au NP-on-nanoplate structures were rinsed with deionized water and dried by N_2_ gas. The same experimental procedure was accomplished using troponin C (TnC, Abcam), troponin T (TnT, Sigma-Aldrich), immunoglobulin G (IgG, Sigma-Aldrich), and avidin (Sigma-Aldrich) instead of cTnI for the selectivity test.

### 2.5. Assays of Clinical Serum Samples

Nine clinical serum samples were collected at Kyung Hee University Hospital from patients with (6 samples) or without (3 samples) AMI. The clinical study was approved by the Institutional Review Board (IRB) of the Kyung Hee University Hospital (KMC IRB 1104-04). Consent documents were obtained from all patients included in this study. All serum samples were stored at −80 °C until use. The concentration of cTnI in each of the clinical serum samples was determined using the SERS-based method and ELISA (ab200016, abcam), respectively.

### 2.6. Instrumentation

SERS spectra were acquired using a homemade micro-Raman system based on an Olympus BX41 microscope (Olympus, Japan). A He-Ne laser with 633 nm radiation (Melles Griot, Australia) was used as the excitation source and the laser light was focused on the sample through a 50× objective (Mitutoyo, Japan) of the microscope. The spectra were measured with a thermodynamically cooled electron-multiplying charge-coupled device (Andor, UK) mounted on the spectrometer with a 1200 groove/mm grating (Dongwoo Optron, Korea). A holographic notch filter was used to reject the laser light (Kaiser Optical Systems, USA). The acquisition time was 1 min. SEM images were obtained from a Nova230 microscope (FEI Company). AFM images were obtained from a Park Systems XE-100 microscope.

## 3. Results and Discussion

**Figure 1:**
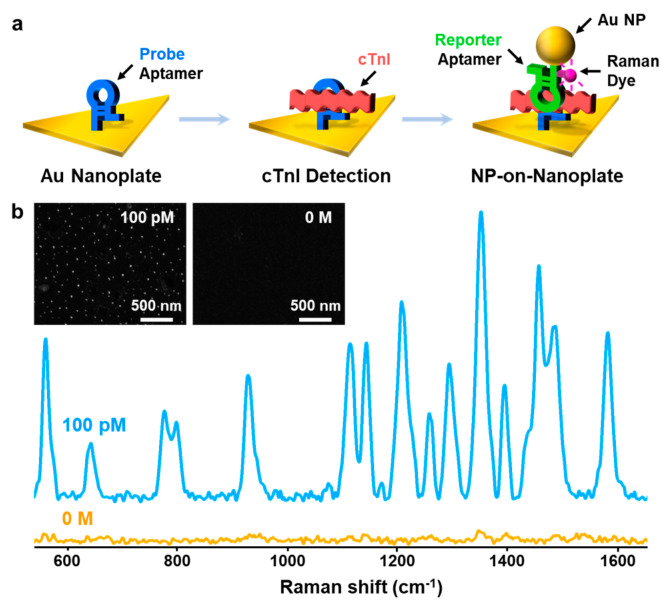
(**a**) Schematic representation of the cardiac troponin I protein (cTnI) detection procedure using an aptamer-immobilized Au nanoplate. (**b**) Surface-enhanced Raman scattering (SERS) spectra of Cy5 measured from Au nanoparticles (NPs)-on-nanoplate structure after detection of cTnI (100 pM, blue spectrum) and the control sample (orange spectrum). The inset shows SEM images of Au NPs-on-nanoplate structures after detection of cTnI (left panel) and the control sample (right panel).

Figure 1a shows a schematic procedure of cTnI detection by using an aptamer-immobilized Au nanoplate. The Au nanoplate was modified with a probe aptamer by incubating and washing. The aptamer-immobilized Au nanoplates were then incubated in a sample solution and washed. Finally, the Au NP reporter solution was reacted with the cTnI-captured nanoplates and washed. When the sample contained cTnIs, the NPs-on-nanoplate architecture could be constructed. Moreover, the cTnI-detected Au NPs-on-Au nanoplate structure can provide strong SERS signals of Cy5 at the nanogaps of NPs and nanoplates because the Au NP reporter includes Cy5. Therefore, we could identify cTnI by measuring the SERS signals.

The atomically flat Au nanoplates were prepared by a vapor transport method (Figure S1a). As-synthesized Au nanoplates have triangular shapes and ~100 nm thicknesses (Figure S1b,c). The length of the nanoplates can be controlled from approximately a few micrometers to tens of micrometers by changing the reaction conditions. Transmission electron microscopy (TEM) analysis indicates that the Au nanoplates are single crystalline with (111) in-plane orientation (Figure S1d–g).

Table 1 shows the aptamers for cTnI. Compared with the K_d_ value of the commercially available antibody against cTnI (20.8 nM) [5], the binding affinities of the probe (3.10 nM) and reporter (3.37 nM) aptamers for cTnIs are stronger. The previous report suggests the non-intersection of binding sites between the probe and reporter aptamers [4,5]. The long complementary sequences of the troponin aptamers can stabilize their secondary structures and improve the binding capabilities for cTnI. Figure S2 shows the predicted secondary structure of the probe aptamer. For the simple immobilization of the probe aptamer onto the Au nanoplates, we added a thiol residue at the 3′-terminus of the probe aptamer. In addition, we added Cy5 at the 5′-terminus and a thiol group at the 3′-terminus of the reporter aptamer. Cy5 has an absorption maximum at 647 nm, allowing incoming light at a wavelength of 633 nm to excite the resonant vibration of Cy5 [25].

Figure 1b is the SERS spectra of Cy5 after the sensing of cTnI (100 pM and control) by using the aptamer-immobilized Au nanoplate platforms. When the 100 pM of cTnI solution (2.4 ng/mL) was used, four major bands of Cy5 at 1580, 1485, 1360, and 1185 cm^−1^, which corresponds to *v*(C=N)_stretch_, *v*(C-C)_ring_, *v*(C=C)_ring_, and *v*(C-N)_stretch_ were clearly observed (blue spectra in Figure 1b) [25]. Because Cy5 was only added to the reporter aptamer, the highly enhanced SERS signals of Cy5 verified the formation of the NPs-on-nanoplate architectures and the corresponding sensing of cTnI. The SEM image of the NPs-on-nanoplate structure also supports the SERS results (left panel in the inset of Figure 1b). When the control sample was employed, very weak SERS signals of Cy5 were obtained (orange spectra in Figure 1b) and the corresponding SEM image shows no Au NPs (right panel in the inset of Figure 1b). This suggests that the aptamer-immobilized Au nanoplate platform can identify cTnI.

**Figure 2:**
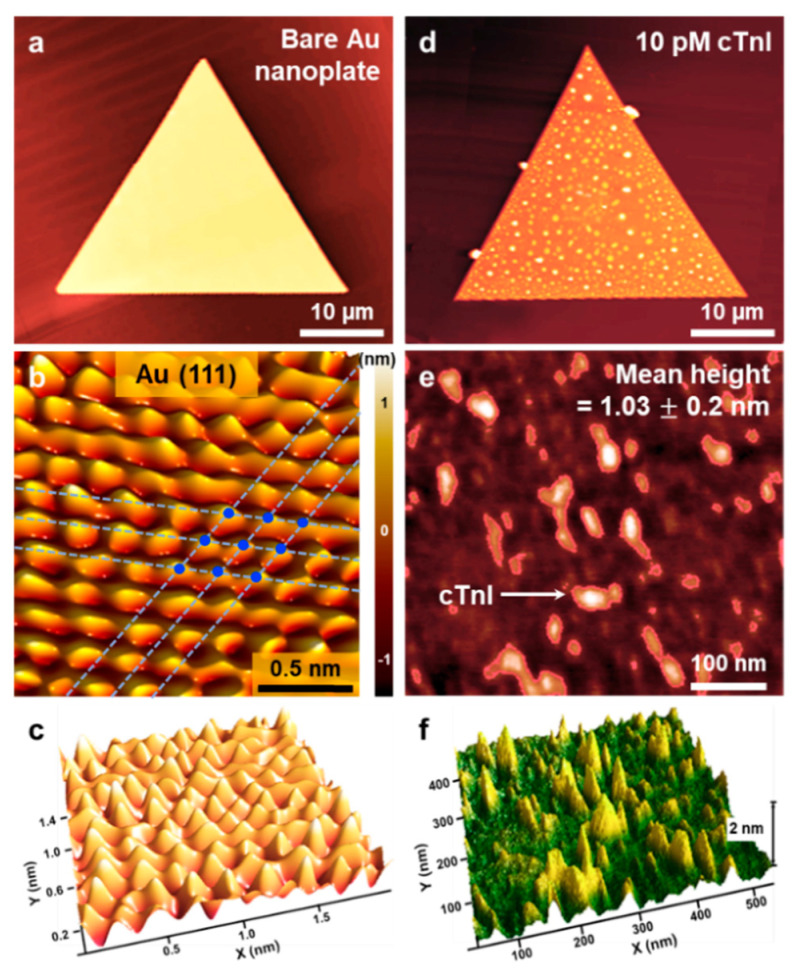
(**a**) Atomic force microscopy (AFM) topography image of a bare Au nanoplate. (**b**) Fourier bandpass-filtered atomic stick-slip image showing a well-defined Au (111) surface. (**c**) Three-dimensional Fourier bandpass-filtered atomic stick-slip image of the Au (111) surface. (**d**) AFM topography image of an aptamer-immobilized Au nanoplate after reaction with cTnI (10 pM). (**e**) Magnified and (**f**) three-dimensional AFM images of a cTnI-captured Au nanoplate.

Figure 2a presents an AFM topography image of a bare Au nanoplate. The root-mean-square (rms) roughness and line-profile roughness of the Au nanoplate were measured as 0.12 nm and −0.2 nm, respectively (Figure S3), indicating an atomically flat Au surface without defects. Figure 2b,c is a high-resolution AFM image of an Au nanoplate, showing atomic stick-slip events clearly. A Fourier bandpass-filtered image of the Au nanoplate displays a 3-fold symmetric atomic stick-slip pattern with a periodicity of 0.28 ± 0.02 nm. The interatomic spacing (0.288 nm) on the Au (111) surface is consistent with the measured value. The AFM results of a bare Au nanoplate clearly confirm that the nanoplates have atomically flat Au (111) surfaces without additional treatments, such as thermal annealing and chemical etching. Moreover, the Au nanoplates were synthesized in the vapor phase without any surfactants or other contaminants, providing substantially reduced surface roughness compared to that obtained with other synthesis methods. 

The large-area formation of atomically flat Au surfaces is important because they can effectively interface with various biological systems. Previously, the combination of Au nanoplates and biological receptors synergistically contributed to the sensitive and selective sensing of molecules [17,18,19]. Therefore, we sought to immobilize the high-performance aptamer for cTnI onto the Au nanoplates and apply the aptamer-immobilized Au nanoplates for diagnosing AMI. Figure 2d presents an AFM image of an aptamer-immobilized Au nanoplate after the detection of cTnI (10 pM). Compared to those on the bare Au nanoplate, cTnIs captured on the whole Au nanoplate were observed. Figure 2e,f and Figure S4 present magnified AFM images. The mean height of 1.03 ± 0.2 nm (10,000 peaks in Figure 2e,f) suggests that the probe aptamer was vertically immobilized and well-ordered. Furthermore, considering the size of the cTnI (width of 2 nm and length of 20 nm), we speculated that the objects in Figure 2e,f and Figure S4 correspond to the captured cTnIs.

**Figure 3:**
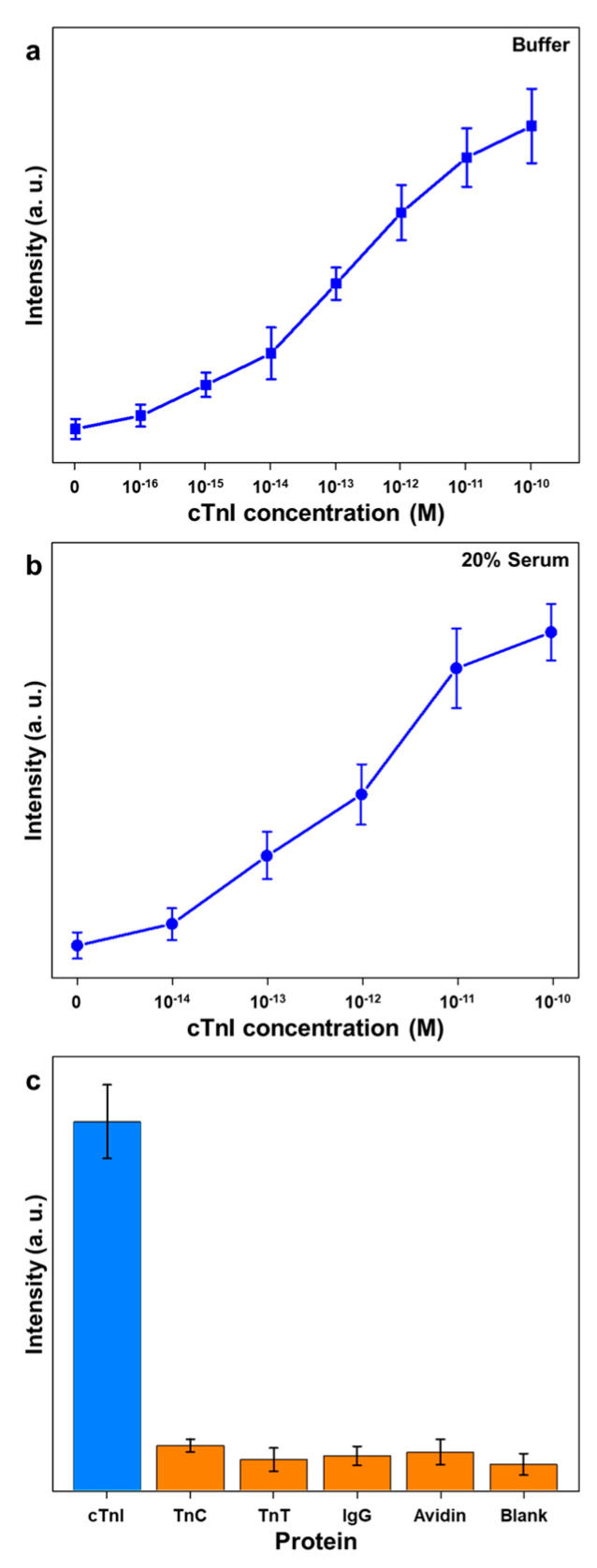
(**a**) Plot of 1580 cm^−1^ band intensity versus cTnI concentration (0–10^−10^ M) in buffer. (**b**) Plot of 1580 cm^−1^ band intensity versus cTnI concentration (0–10^−10^ M) in 20% serum. (**c**) Plot of 1580 cm^−1^ band intensity versus proteins (cTnI, TnC, TnT, IgG, avidin, and control sample). The data represent the average plus standard deviation from ten measurements.

For the quantitative detection of cTnI, the aptamer-immobilized Au nanoplates were reacted with a variety of concentrated cTnI solutions and the Au NP reporters. We obtained the SERS signals from ten randomly selected samples. Figure 3a shows a plot of the 1580 cm^−1^ band intensity versus the cTnI concentration in the buffer. The full SERS spectra are presented in Figure S5. The intensity gradually increased as the concentration of cTnI increased. When the control sample was used, the SERS spectra were barely detectable. This finding suggests that the nonspecific bindings of Au NP reporters were reduced by the atomically flat Au nanoplates. When a 10 aM of cTnI solution (240 aM/mL) was employed, the SERS signals were undistinguishable from the signals of the control sample (data not shown). We obtained distinguishable SERS signals when the sample contained 100 aM of cTnI (2.4 fg/mL). This demonstrates that the aptamer-immobilized Au nanoplate platform can detect cTnI at the attomolar level. Notably, the detection limit of the present method is significantly lower than previously reported methods (Table S1). This ultrasensitivity might be due to the strong binding affinity of the aptamer with cTnI and the uniform immobilization of the aptamer on the atomically flat Au nanoplates.

In a typical hospital setting, after a blood separation process, serum is employed for the diagnosis of AMI. Therefore, cTnI-sensing methods for the diagnosis of AMI should operate effectively in serum. We examined the present method to detect cTnI in serum. Figure 3b is a plot of the 1580 cm^−1^ band intensity versus the cTnI concentration in 20% serum. The full SERS spectra are shown in Figure S6. When a control sample was employed, very weak but distinguishable SERS signals of Cy5 were obtained. This result might be attributed to the nonspecific binding of proteins in the serum and Au NP reporters. Because serum contains several kinds of proteins, electrolytes, hormones, and exogenous substances, nonspecific bindings could be induced. As shown in Figure 3b, the SERS signals increased from the cTnI concentration of 10^−14^ to 10^−11^ M gradually. This observation indicates that the aptamer-immobilized Au nanoplate platform enabled us to detect cTnI in 20% human serum at 100 fM (2.4 pg/mL).

We further investigated the selective detection of cTnI using the aptamer-immobilized Au nanoplate platform. Figure 3c displays a plot of the 1580 cm^−1^ band intensities for several kinds of proteins (cTnI, TnC, TnT, IgG, avidin, and control). The concentrations of all proteins in the buffer were 10 pM. As shown in Figure 3c, strong SERS signals of Cy5 were obtained (blue bar) only in the presence of cTnI. In the presence of TnC, TnT, IgG, and avidin, negligible SERS signals were obtained (orange bars). This result suggests that the aptamer-immobilized Au nanoplates can identify cTnI specifically. Although the SERS signals of nontarget proteins are very weak, they are distinguished from the signals of blank samples. In serum, IgG (5.6–18 g/L) and albumin (35–50 g/L) are present in high concentrations [26]. Consequently, the proteins in serum may induce the background SERS signals. The difference of detection limits in the buffer and serum can interfere with the practical use of the current method. Nevertheless, we speculate that the detection limit of 100 fM (2.4 pg/mL) shows potential in the practical detection of cTnI in serum.

**Figure 4:**
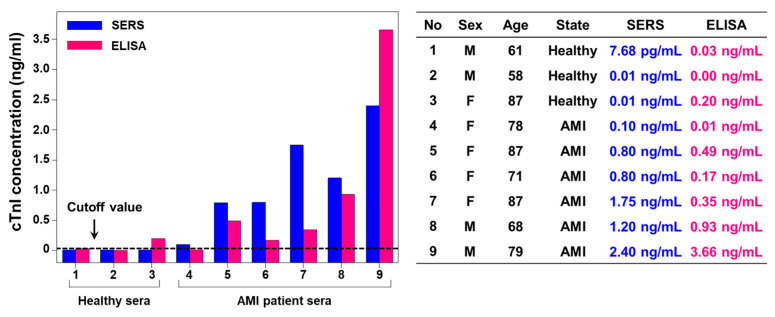
Diagnostic result for clinical samples based on SERS and ELISA data.

Finally, the aptamer-immobilized Au nanoplate platform was applied to the clinical diagnosis of AMI. Nine clinical samples (three samples from healthy people and six samples from AMI patients) were collected from Kyung Hee University Medical Center and handled in accordance with approved IRB protocols at the hospital. The sex ratio of the samples was 4:5 (male:female), and the average age of the patients was 74.3, with a maximum of 87 and a minimum of 58. Figure 4 presents the diagnostic results for the clinical samples based on the SERS and ELISA data. For the SERS assay, the data in Figure 3b were employed as a calibration curve. Although the cutoff levels of cTnI vary according to the patients and detection methods, it is known that the 99th percentile and clinical cutoff levels for cTnI are 70 and 400 pg/mL, respectively [4]. The cTnI concentrations measured from the NP-on-nanoplate structures were consistent with those measured from ELISA within the clinically acceptable range. Both methods showed relatively low cTnI concentrations for healthy people but high cTnI concentrations for AMI patients. The dynamic ranges of SERS and ELISA methods are 2.4 pg/mL–2.4 ng/mL and 30 pg/mL–4 ng/mL, respectively. Therefore, the developed SERS approach may provide the accurate diagnostic results in low concentrated cTnI samples. Note that use of this method for diagnosing AMI patients in the real world should be thoroughly tested. After careful validation, the cutoff value of our method might be determined exactly.

Although many biochemical parameters of heart-tissue origin have been used in the diagnosis of AMI from past to present, there is no consensus on the best cardiac biomarker [1]. Analysis of a single biomarker is not recommended since there is no single ideal and specific biomarker [1]. Currently, cTnI and cardiac troponin T protein (cTnT) are the gold-standard biomarkers for diagnosing AMI [3]. Additionally, other candidate biomarkers, such as heart-type fatty acid binding protein (hFABP), glycogen phosphorylase isoenzyme BB (GPBB), S100, pregnancy associated plasma protein-A (PAPP-A), C-reactive protein (CRP), tumor necrosis factor (TNF), interleukin 6 (IL6), interleukin 18 (IL18), CD40 ligand, myeloperoxidase (MPO), matrix metalloproteinase-9 (MMP-9), cell-adhesion molecules, oxidized low-density lipoprotein (LDL), glutathione, homocysteine, fibrinogen, and D-dimer procalcitonin, may also play a role in the diagnosis of AMI [1,3]. The current method demonstrated cTnI detection only, however, we anticipate that the method will be used for the multiplex detection of various AMI biomarkers and thus will allow a more accurate diagnosis of AMI.

## 4. Conclusions

We report that an optimal aptamer-immobilized Au nanoplate platform enabled the sensitive and selective sensing of cTnI. The current study is important for the following reasons. First, the aptamer-immobilized Au nanoplate platform can detect cTnIs in the buffer at a low concentration of 100 aM (2.4 fg/mL). Compared to the previously reported cTnI sensing methods, the detection limit of this method is lower. Second, the high sensitivity of the aptamer-immobilized Au nanoplate platform can be maintained in serum tests. cTnIs in serum can be identified at a low concentration of 100 fM (2.4 pg/mL). This detection limit is much lower than the existing cutoff values, suggesting the potential diagnostic ability of the method. Third, the aptamer-immobilized Au nanoplate allowed us to diagnose nine clinical samples more accurately than conventional ELISA. In this regard, we expect that uniformly immobilized bioreceptors on Au nanoplates will be useful for the diagnosis of several diseases.

## Figures and Tables

**Table 1:** Aptamer sequences used in this experiment.

Aptamer	Sequence	K_d_ (cTnI)	K_d_ (Troponin Complex)
Probe	5′-CGCATGCCAAACGTTGCCTCATAGT TCCCTCCCCGTGTCC-HS-(CH_2_)_6_-3′	317 pM	3.37 nM
Reporter	5′-Cy5-CGTGCAGTACGCCAACCTTTCTCA TGCGCTGCCCCTCTTA-HS-(CH_2_)_6_-3′	270 pM	3.10 nM

## Supplementary Materials

The following are available online at https://www.mdpi.com/2079-4991/10/7/1402/s1, Figure S1: Schematic illustration of the experimental setup for the synthesis of Au nanoparticles and electron microscope images of Au nanoplates, Figure S2: Predicted secondary structure of probe aptamer, Figure S3: AFM analysis data of an Au nanoplate, Figure S4: AFM image of an aptamer-immobilized Au nanoplate after detection of cTnI, Figure S5: Full SERS spectra corresponding to Figure 3a, Figure S6: Full SERS spectra corresponding to Figure 3b: Table S1: Comparison of other cTnI detection methods.

## References

[1] Aydin S., Ugur K., Aydin S., Sahin I., Yardim M. (2019). Biomarkers in acute myocardial infarction: Current perspectives. Vasc. Health Risk Manag..

[2] Benjamin E.J., Blaha M.J., Chiuve S.E., Cushman M., Das S.R., Deo R., de Ferranti S.D., Floyd J., Fornage M., Gillespie C. (2017). Heart Disease and Stroke Statistics—2017 Update. Circulation.

[3] Soetkamp D., Raedschelders K., Mastali M., Sobhani K., Bairey-Merz C.N., Van Eyk J. (2017). The continuing evolution of cardiac troponin I biomarker analysis: From protein to proteoform. Expert Rev. Proteomics.

[4] Jo H., Gu H., Jeon W., Youn H., Her J., Kim S.-K., Lee J., Shin J.H., Ban C. (2015). Electrochemical Aptasensor of Cardiac Troponin I for the Early Diagnosis of Acute Myocardial Infarction. Anal. Chem..

[5] Jo H., Her J., Lee H., Shim Y.-B., Ban C. (2017). Highly sensitive amperometric detection of cardiac troponin I usingsandwich aptamers and screen-printed carbon electrodes. Talanta.

[6] Cho J.-H., Han S.-M., Paek E.-H., Cho I.-H., Paek S.-H. (2006). Plastic ELISA-on-a-Chip Based on Sequential Cross-Flow Chromatography. Anal. Chem..

[7] Cummins B., Auckland M.L., Cummins P. (1987). Cardiac-specific troponin-I radioimmunoassay in the diagnosis of acute myocardial infarction. Am. Heart J..

[8] Song S.Y., Han Y.D., Kim K., Yang S.S., Yoon H.C. (2011). A fluoro-microbead guiding chip for simple and quantifiable immunoassay of cardiac troponin I (cTnI). Biosens. Bioelectron..

[9] Hemming E., Temiz Y., Gökçe O., Lovchik R.D., Delamarche E. (2020). Transposing Lateral Flow Immunoassays to Capillary-Driven Microfluidics Using Self-Coalescence Modules and Capillary-Assembled Receptor Carriers. Anal. Chem..

[10] Cheng Z., Wang R., Xing Y., Zhao L., Choo J., Yu F. (2019). SERS-based immunoassay using gold-patterned array chips for rapid and sensitive detection of dual cardiac biomarkers. Analyst.

[11] Fu X., Wang Y., Liu Y., Liu H., Fu L., Wen J., Li J., Wei P., Chen L. (2019). A graphene oxide/gold nanoparticle-based amplification method for SERS immunoassay of cardiac troponin I. Analyst.

[12] Lv H., Li Y., Zhang X., Li X., Xu Z., Chen L., Li D., Dong Y. (2019). Thionin functionalized signal amplification label derived dual-mode electrochemical immunoassay for sensitive detection of cardiac troponin I. Biosens. Bioelectron..

[13] Bai T., Wang M., Cao M., Zhang J., Zhang K., Zhou P., Liu Z., Liu Y., Guo Z., Lu X. (2018). Functionalized Au@ Ag-Au nanoparticles as an optical and SERS dual probe for lateral flow sensing. Anal. Bioanal. Chem..

[14] Chon H., Lee S., Yoon S.Y., Lee E.K., Chang S.I., Choo J. (2014). SERS-based competitive immunoassay of troponin I and CK-MB markers for early diagnosis of acute myocardial infarction. Chem. Commun..

[15] Khlebtsov B.N., Bratashov D.N., Byzova N.A., Dzantiev B.B., Khlebtsov N.G. (2019). SERS-based lateral flow immunoassay of troponin I by using gap-enhanced Raman tags. Nano Res..

[16] Zhang D., Huang L., Liu B., Ni H., Sun L., Su E., Chen H., Gu Z., Zhao X. (2018). Quantitative and ultrasensitive detection of multiplex cardiac biomarkers in lateral flow assay with core-shell SERS nanotags. Biosens. Bioelectron..

[17] Yoo Y., Lee H., Lee H., Lee M., Yang S., Hwang A., Kim S., Park J.Y., Choo J., Kang T. (2017). Surfactant-Free Vapor-Phase Synthesis of Single-Crystalline Gold Nanoplates for Optimally Bioactive Surfaces. Chem. Mater..

[18] Lee M., Kim H., Kim E., Yi S.Y., Hwang S.G., Yang S., Lim E.-K., Kim B., Jung J., Kang T. (2018). Multivalent Antibody-Nanoparticle Conjugates To Enhance the Sensitivity of Surface-Enhanced Raman Scattering-based Immunoassays. ACS Appl. Mater. Interfaces.

[19] Hwang A., Kim E., Moon J., Lee H., Lee M., Jeong J., Lim E.-K., Jung J., Kang T., Kim B. (2019). Atomically Flat Au Nanoplate Platforms Enable Ultraspecific Attomolar Detection of Protein Biomarkers. ACS Appl. Mater. Interfaces.

[20] Kang T., Yoo S.M., Yoon I., Lee S.Y., Kim B. (2010). Patterned Multiplex Pathogen DNA Detection by Au Particle-on-Wire SERS Sensor. Nano Lett..

[21] Lee H., Jeong K.-Y., Kang T., Seo M.-K., Kim B. (2014). A twin-free single-crystal Ag nanoplate plasmonic platform: Hybridization of the optical nano-antenna and surface plasmon active surface. Nanoscale.

[22] Guk K., Kim H., Lee M., Choi Y.-A., Hwang S.G., Han G., Kim H.-N., Kim H., Park H., Yong D. (2020). Development of Novel A4 Antibody for Detection of Neuraminidase I223R/H275Y-Associated Antiviral Multidrug-Resistant Influenza Virus. Nat. Commun..

[23] Roy D., Park J.W. (2015). Spatially Nanoscale-controlled Functional Surfaces toward Efficient Bioactive Platforms. J. Mater. Chem. B.

[24] Jarczewska M., Górski Ł., Malinowska E. (2016). Electrochemical aptamer-based biosensors as potential tools for clinical diagnostics. Anal. Methods.

[25] Kang T., Yoo S.M., Yoon I., Lee S., Choo J., Lee S.Y., Kim B. (2011). Au Nanowire-on-Film SERRS Sensor for Ultrasensitive Hg^2+^ Detection. Chem. Eur. J..

[26] Jazayeri M.H., Pourfathollah A.P., Rasaee M.J., Porpak Z., Jafari M.E. (2013). The concentration of total serum IgG and IgM in sera of healthy individuals varies at different age intervals. Biomed. Aging Pathol..

