# Supplementary material for: Troponin Aptamer on an Atomically Flat Au Nanoplate Platform for Detection of Cardiac Troponin I
Source: Nanomaterials (Basel). 2020 Jul 18;10(7):1402. doi: 10.3390/nano10071402 (PMC7407982; doi:10.3390/nano10071402)
Supplement: Supplementary file 1 [file nanomaterials-10-01402-s001.pdf]

*nanomaterials*

Supporting Information

# Troponin Aptamer on an Atomically Flat Au Nanoplate Platform for Detection of Cardiac Troponin I

**Hyoban Lee [1], Hyungjun Youn [2], Ahreum Hwang [1,3], Hyunsoo Lee [1,4], Jeong Young Park [1,4], Weon Kim [5], Youngdong Yoo [6], Changill Ban [2,*], Taejoon Kang [3,*] and Bongsoo Kim [1,*]**

1 Department of Chemistry, KAIST, Daejeon 34141, Korea; stban1829@gmail.com (H.L.); ahreumh@kaist.ac.kr (A.H.); hsoolee@kaist.ac.kr (H.L.); jeongypark@kaist.ac.kr (J.Y.P.)
2 Department of Chemistry, POSTECH, Pohang 37673, Korea; yhj1005@postech.ac.kr
3 Bionanotechnology Research Center, KRIBB, Daejeon 34141, Korea
4 Center for Nanomaterials and Chemical Reactions, IBS, Daejeon 34141, Korea
5 Division of Cardiology, Department of Internal Medicine, Kyung Hee University Hospital, Kyung Hee University, Seoul 02447, Korea; mylovekw@hanmail.net
6 Department of Chemistry, Ajou University, Suwon 16499, Korea; yyoo@ajou.ac.kr
* Correspondence: bongsoo@kaist.ac.kr (B.K.); kangtaejoon@kribb.re.kr (T.K.); ciban@postech.ac.kr (C.B.)

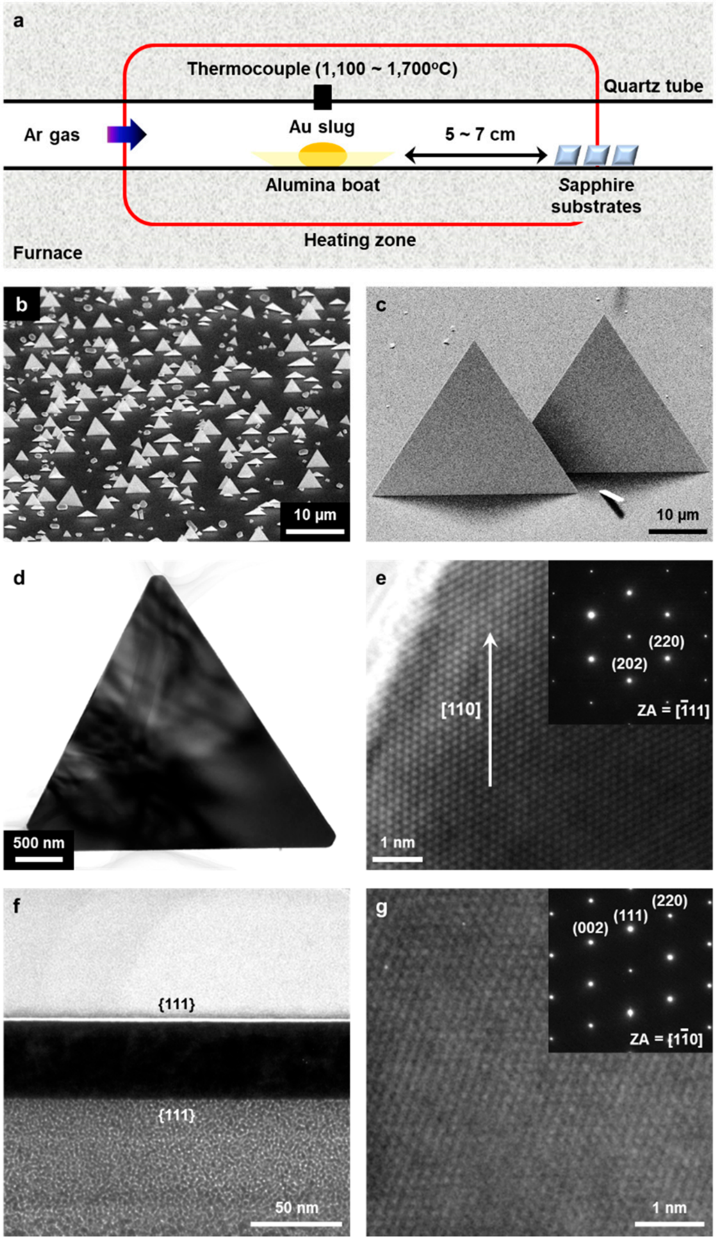


**Figure S1.** (**a**) Schematic illustration of the experimental setup for the synthesis of Au nanoparticles. (**b, c**) SEM images of Au nanoplates on a sapphire substrate. (**d**) TEM image of the Au nanoplate. (**e**) High-resolution transmission electron microscopy (HRTEM) image of the Au nanoplate. Inset is a selected area electron diffraction (SAED) pattern of the Au nanoplate. (**f**) Cross-sectional TEM image of Au nanoplate. (**g**) HRTEM image and SAED pattern of Au nanoplate.

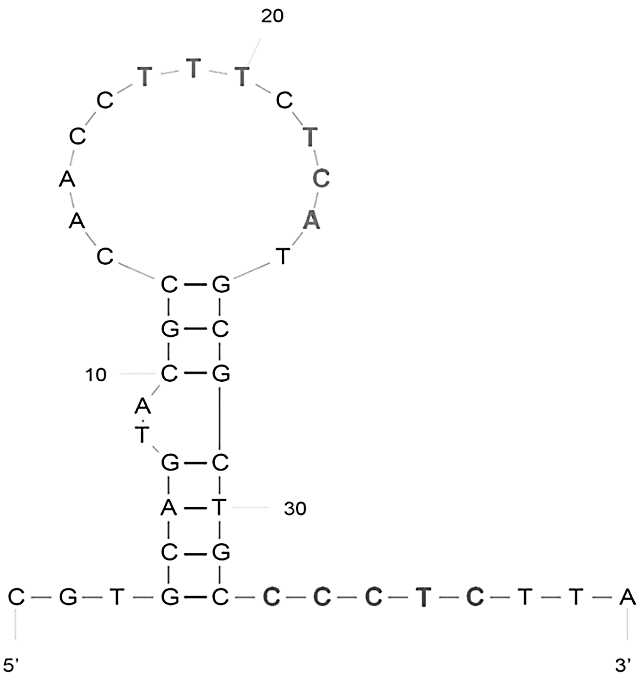


**Figure S2.** Predicted secondary structure of probe aptamer..

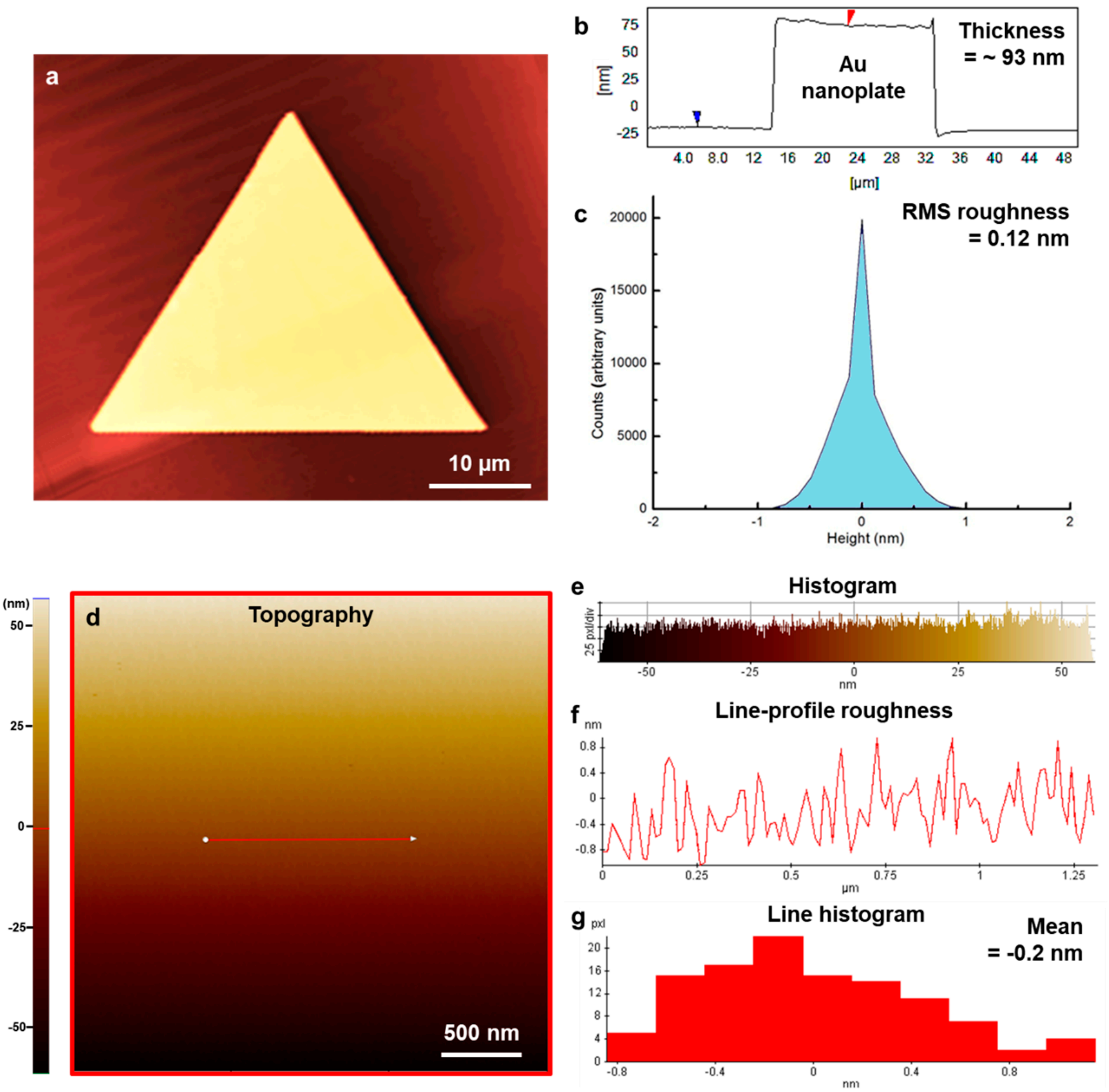


**h** Statistics

| Line | Min(nm) | Max(nm) | Mid(nm) | Mean(nm) | Rpv(nm) | Rq(nm) | Ra(nm) | Rz(nm) | Rsk | Rku |
|---|---|---|---|---|---|---|---|---|---|---|
| Red | -1.041 | 0.946 | -0.048 | -0.209 | 1.986 | 0.450 | 0.367 | 1.754 | -0.406 | 2.640 |

**Figure S3.** (**a**) AFM topography image of an atomically flat Au nanoplate. (**b**) Sectional view of the Au nanoplate ($R_q$ = 0.15 nm). (**c**) Surface-height distribution of the Au nanoplate ($S_q$ = 0.15 nm). (**d**) Magnified AFM topography image of the Au nanoplate. (**e-h**) Histogram (e), line-profile roughness (f), line histogram (g), and statistics (g) obtained from the red line in (d).

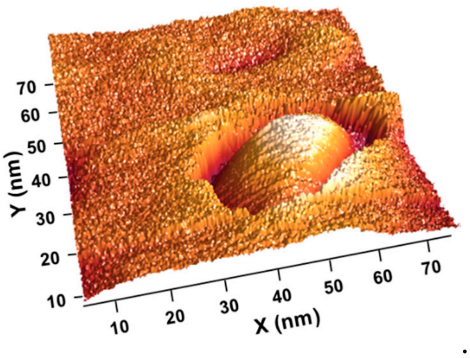


**Figure S4.** Three-dimensional AFM image of an aptamer-immobilized Au nanoplate after reaction with cTnI.

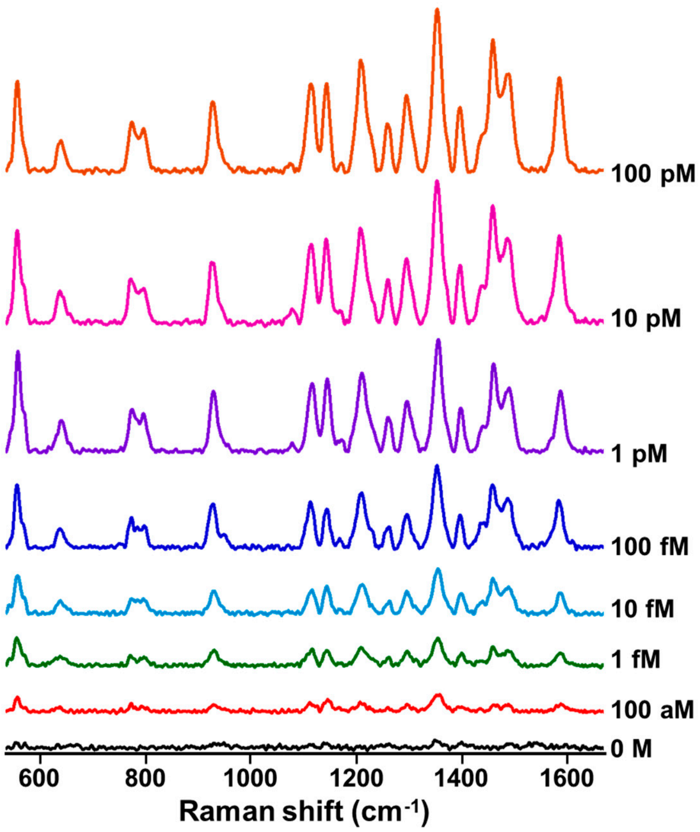


**Figure S5.** Full SERS spectra corresponding to Figure 3a.

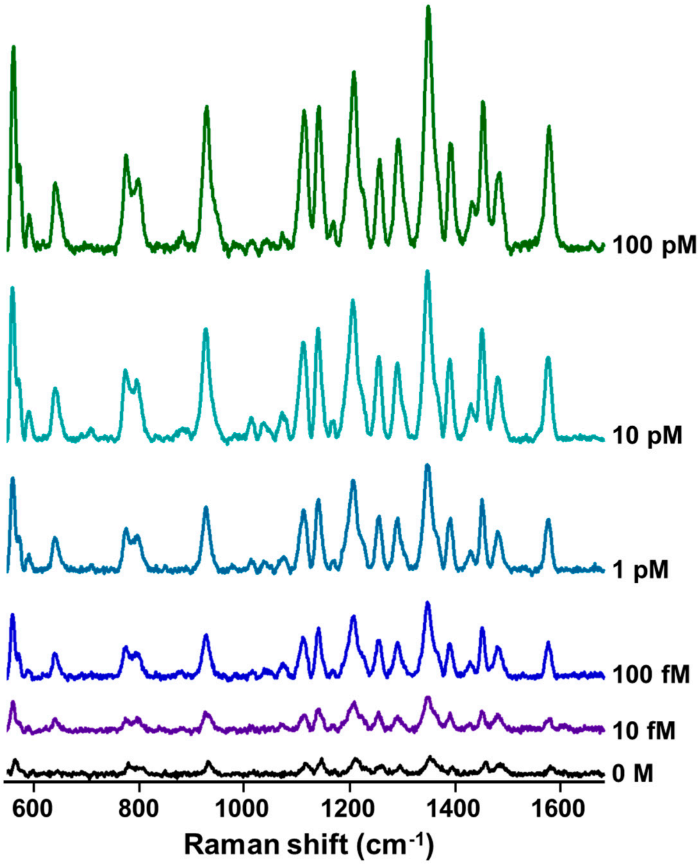


**Figure S6.** Full SERS spectra corresponding to Figure 3b.

**Table S1.** Comparison of other cTnI detection methods.

| Signal | Detection Limit | Tested Matrix | Reference |
|---|---|---|---|
| Electrochemistry | 1.0 pM | Buffer | [1] |
| Electrochemistry | 700 aM | Buffer | [2] |
| Amperometry | 1.0 pM | Serum | [3] |
| Fluorescence | 3.4 pM | Plasma | [4] |
| Fluorescence | 167 nM | Serum | [5] |
| SERS | 372 fM | Buffer | [6] |
| SERS | 210 fM | Buffer | [7] |
| SERS | 3.76 pM | Serum | [8] |
| SERS | 1.41 pM | Buffer | [9] |
| SERS | 4.18 pM | Buffer | [10] |
| SERS | 33.4 pM | Buffer | [11] |
| SERS | 100 aM | Buffer | This work |
| | 100 fM | Serum | |

## References


1. Jo, H.; Gu, H.; Jeon, W.; Youn, H.; Her, J.; Kim, S.-K.; Lee, J.; Shin, J. H.; Ban, C. Electrochemical Aptasensor of Cardiac Troponin I for the Early Diagnosis of Acute Myocardial Infarction. *Anal. Chem.* **2015**, *87*, 9869-9875.
2. Lv. H.; Li, Y.; Zhang, X.; Li, X.; Xu, Z.; Chen, L.; Li, D.; Dong, Y. Thionin functionalized signal amplification label derived dual-mode electrochemical immunoassay for sensitive detection of cardiac troponin I. *Biosens. Bioelectron*. **2019**, *133*, 72-78.
3. Jo, H.; Her, J.; Lee, H.; Shim, Y.-B.; Ban, C. Highly sensitive amperometric detection of cardiac troponin I usingsandwich aptamers and screen-printed carbon electrodes. *Talanta* **2017**, *165*, 442-448.
4. Song, S. Y.; Han, Y. D.; Kim, K.; Yang, S. S.; Yoon, H. C. A fluoro-microbead guiding chip for simple and quantifiable immunoassay of cardiac troponin I (cTnI). *Biosens. Bioelectron.* **2011**, *26*, 3818-3824.
5. Hemming, E.; Temiz, Y.; Gökçe, O.; Lovchik, R. D.; Delamarche, E. Transposing Lateral Flow Immunoassays to Capillary-Driven Microfluidics Using Self-Coalescence Modules and Capillary-Assembled Receptor Carriers. *Anal. Chem.* **2020**, *92*, 940-946.
6. Cheng, Z.; Wang, R.; Xing, Y.; Zhao, L.; Choo, J.; Yu, F. SERS-based immunoassay using gold-patterned array chips for rapid and sensitive detection of dual cardiac biomarkers. *Analyst*, **2019**, *144*, 6533-6540.
7. Fu, X.; Wang, Y.; Liu, Y.; Liu, H.; Fu, L.; Wen, J.; Li, J.; Wei, P.; Chen, L. A graphene oxide/gold nanoparticle-based amplification method for SERS immunoassay of cardiac troponin I. *Analyst*, **2019**, *144*, 1582-1589.
8. Bai, T.; Wang, M.; Cao, M.; Zhang, J.; Zhang, K.; Zhou, P.; Liu, Z.; Liu, Y.; Guo, Z.; Lu, X. Functionalized Au@ Ag-Au nanoparticles as an optical and SERS dual probe for lateral flow sensing. *Anal. Bioanal. Chem*., **2018**, *410*, 2291-2303.
9. Chon, H.; Lee, S.; Yoon, S.Y.; Lee, E.K.; Chang, S.I; Choo, J.; SERS-based competitive immunoassay of troponin I and CK-MB markers for early diagnosis of acute myocardial infarction. *Chem. Commun.* **2014**, *50*, 1058-1060.
10. Khlebtsov, B.N.; Bratashov, D.N.; Byzova, N.A.; Dzantiev, B.B.; Khlebtsov, N.G.; SERS-based lateral flow immunoassay of troponin I by using gap-enhanced Raman tags. *Nano Res.* **2019**, *12*, 413-420.
11. Zhang, D.; Huang, L.; Liu, B.; Ni, H.; Sun, L.; Su, E.; Chen, H.; Gu, Z.; Zhao, X.; Quantitative and ultrasensitive detection of multiplex cardiac biomarkers in lateral flow assay with core-shell SERS nanotags. *Biosens. Bioelectron.* **2018**, *106*, 204-211.
